# Decoding intracranial EEG data with multiple kernel learning method

**DOI:** 10.1016/j.jneumeth.2015.11.028

**Published:** 2016-03-01

**Authors:** Jessica Schrouff, Janaina Mourão-Miranda, Christophe Phillips, Josef Parvizi

**Affiliations:** a Laboratory of Behavioral & Cognitive Neuroscience, Stanford Human Intracranial Cognitive Electrophysiology Program (SHICEP), Department of Neurology & Neurological Sciences, Stanford University, Stanford, CA, USA; b Department of Computer Science, University College London, United Kingdom; c Cyclotron Research Centre, University of Liège, Belgium

**Keywords:** Multiple kernel learning, Intracranial EEG, Machine learning, Feature selection

## Abstract

**Background:**

Machine learning models have been successfully applied to neuroimaging data to make predictions about behavioral and cognitive states of interest. While these multivariate methods have greatly advanced the field of neuroimaging, their application to electrophysiological data has been less common especially in the analysis of human intracranial electroencephalography (iEEG, also known as electrocorticography or ECoG) data, which contains a rich spectrum of signals recorded from a relatively high number of recording sites.

**New method:**

In the present work, we introduce a novel approach to determine the contribution of different bandwidths of EEG signal in different recording sites across different experimental conditions using the *Multiple Kernel Learning* (MKL) method.

**Comparison with existing method:**

To validate and compare the usefulness of our approach, we applied this method to an ECoG dataset that was previously analysed and published with univariate methods.

**Results:**

Our findings proved the usefulness of the MKL method in detecting changes in the power of various frequency bands during a given task and selecting automatically the most contributory signal in the most contributory site(s) of recording.

**Conclusions:**

With a single computation, the contribution of each frequency band in each recording site in the estimated multivariate model can be highlighted, which then allows formulation of hypotheses that can be tested *a posteriori* with univariate methods if needed.

## Highlights

• Multiple Kernel Learning (MKL) can explore multiple dimensions simultaneously.
• MKL method is sparse and performs feature selection during modeling of the data.
• We propose to use the MKL method to analyze electrophysiology data (EEG or MEG).
• We provide a prototype example of how MKL method can apply to ECoG data.
• We show multiple dimensions of ECoG signal can contribute to numerical processing.

## 1 Introduction

Intracranial EEG (a.k.a., electrocorticography, ECoG) is the recording of brain's electrical activity with intracranial sensors. Such signals contain information distributed over multiple dimensions, i.e., the spatial (recording sites), the temporal (sampling rate in 1000 samples/s) and frequency domains (0.1–300 Hz). In the current practice, ECoG signals are primarily analyzed using univariate methods, e.g., to detect changes in the power of the electrophysiological activity during a given task, in a specific recording site of interest and bandwidth, which is often the high-frequency broadband (HFB, 50–200 Hz). A primary motivation for the univariate analysis of HFB in a specific brain region is the extent evidence that HFB provides a precise measure of local cortical engagement during a given task (Chang et al., 2010, Flinker et al., 2011, Manning et al., 2009, Mukamel, 2005, Nir et al., 2007, Pasley et al., 2012, Ray et al., 2008, Ray and Maunsell, 2011). Although these univariate studies have a crucial role in testing hypotheses about the local engagement of a specific cortical region in a given task, a more sensitive, data-driven multivariate approach is needed to explore the contribution of many dimensions of the intracranial electrophysiological signal.

Towards establishing a multivariate approach in the analysis of ECoG data, we took advantage of recent machine learning based models that have been applied to other biological data (see (Pereira et al., 2009) for a review). These methods detect regularities/patterns in the signal that can be used to make predictions about new/unseen input data. Due to their multivariate nature, machine learning based techniques are thought to be more sensitive in detecting distributed patterns of brain activity than univariate analyses. They have already proven useful when investigating local field potentials and spikes in both monkeys and humans (e.g., Meyers et al., 2008, Meyers et al., 2012, Quian Quiroga and Panzeri, 2009, Zhang et al., 2011b). However, model interpretation might be complex as the obtained model parameters cannot be thresholded based on their amplitude/variance ratio.

In our current work, we present a Multiple Kernel Learning (MKL) approach to facilitate machine learning modeling of ECoG data. The MKL method simultaneously learns and combines different models, represented by different kernels (see Gönen and Alpaydin, 2011 for a review). MKL approaches have been previously applied to neuroimaging data, especially for combination of signals from different modalities (e.g., Dai et al., 2012, Filipovych et al., 2011, Filippone et al., 2012, Hinrichs et al., 2011, Zhang et al., 2011a). Furthermore, the contribution of each kernel to the final model can be constrained to be sparse (Rakotomamonjy et al., 2008). In the present work, each kernel is built from different features of the ECoG data, i.e., the power of activity in specific bandwidths of ECoG signal at each recording site. MKL is therefore used here as a tool for *feature selection*.

As a proof of concept study, we examined the feasibility of the MKL approach by applying this method to previously published human electrophysiological data (Dastjerdi et al., 2013, Foster et al., 2012). Here we show that with a single computation, the MKL model is able to highlight the contribution of each frequency band at each recording site, which then can be used to formulate hypotheses to be tested *a posteriori*.

## 2 Materials and methods

The current work aims at demonstrating the feasibility of MKL techniques for the analysis of ECoG data. To this end, we considered data that has been previously analyzed with univariate methods (Dastjerdi et al., 2013, Foster et al., 2012). The results of these works showed (1) increased HFB power in the retrosplenial cortex (RSC) during episodic memory processing and inactivation (i.e., no changes in HFB power) of the same recording sites during numerical processing, and (2) increased HFB power in the intra-parietal sulcus (IPS) during numerical processing and deactivation (i.e., decreased HFB power) in the same recording site during episodic memory processing. The present study probes the same dataset in a more exploratory way by considering all recording sites and frequency bands during numerical and episodic memory processing in the same model.

### 2.1 Demographics and brain coverage

Three subjects were implanted with intracranial electrodes for presurgical epilepsy monitoring. All three patients suffered from medication resistant epilepsy for which they were implanted with intracranial electrodes for invasive video EEG monitoring. The procedure was approved by the Stanford Institutional Review Board (IRB) and the subjects provided written informed consent to participate in the study. The location of the grids was determined by clinical needs, and the seizure foci were found to be in the right medial parietal (P1), right medial occipital (P2) and left insular cortices (P3). These areas were resected after electrode explantation in patients P1 and P3. Signal was continuously recorded for 7–10 days during which simultaneous video monitoring was performed (Nihon Kohden Technology, sampling rate: 1000 Hz for P1 and P2, 500 Hz for P3). Electrodes were platinum plates (2.3 mm diameter) with a center-to-center interelectrode spacing of 10 mm. Electrodes containing artifacts were identified by visual inspection of the signal and discarded from further analyses. Electrodes suspected to contain pathological activity – as identified by the clinical team – were also discarded from further analyses. This last step was performed to ensure that non-pathological neuronal activity was used to investigate our cognitive neuroscience question.

### 2.2 Anatomical localization of electrodes

Post-implant CT images were aligned to the pre-op MRI anatomical brain volume (Hermes et al., 2010). Electrodes were visualized on the subject's own brain volume and reconstructed 3D cortical surface allowing for accurate anatomical localization of electrodes.

### 2.3 ECoG experiment

**Fig. 1:**
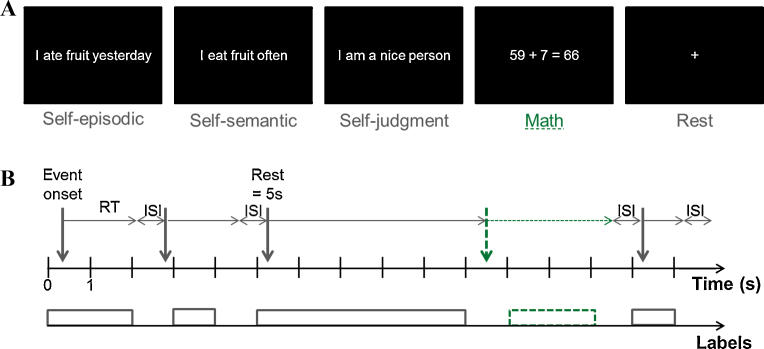
Experimental design and feature extraction. (A) Examples of stimuli of each category. The patient has to provide ‘true’ or ‘false’ judgments on the presented stimulus, except for the ‘Rest’ condition (fixation cross, presented for 5 s). The number of trials per condition is 48 in each run (except for ‘Rest’, 33 trials). In the present work, ‘Math’ is the condition of interest. The four other conditions (namely self-episodic, self-semantic, self-judgment and rest) are pooled together to form the ‘Non-math’ category. (B) Definition of “Events”: The event onset (displayed by vertical arrows) represents the beginning of visual stimulus presentation. Each stimulus is displayed on the computer screen until the participant presses a button (1 for ‘true’ or 2 for ‘false’), in a self-paced manner with varying lengths of reaction time (RT). The next stimulus is then displayed, after an inter-stimulus interval (ISI) of 200 ms. ‘Rest’ condition had fixed intervals and the next stimulus appeared without the participant pressing any buttons. In this work, the continuous time series was divided into 1 s windows. Each window was associated to a label (‘Math’ or ‘Non-math’) if a stimulus was the only stimulus present during the selected 1 s window (bottom line, labels). To avoid including non-task related signal, the event onset was rounded toward the nearest integer and the event duration (here corresponding to the RT) was rounded toward negative infinity (i.e., Matlab ‘floor’).

Data was recorded when the patients performed simple true/false judgments of memory sentences or mathematical equations (Fig. 1A). The memory sentences comprised self-episodic (e.g., “I ate pizza this week”), self-semantic (e.g., “I eat pizza often”) and self-judgment (e.g., “I am a curious person”) statements. Basic mathematical additions were presented along with a result (e.g., “4 + 49 = 53”, further referred to as ‘Math’ condition). Interleaved across trials were 5 s cued-rest periods, during which a centered cross sign was displayed on the screen and patients were instructed to fixate and rest. The experiment comprised 96 randomized trials of each condition (except for rest, ∼66 trials) and was divided in two sessions administered the same day (P1) or on successive days (P2, P3).

### 2.4 Preprocessing

All preprocessing steps were performed using Matlab (www.mathworks.com) and SPM (www.fil.ion.ucl.ac.uk/spm). The continuous time series of the two experimental sessions were first re-referenced to the average of the signals over all selected electrodes (considering each session separately). The data was then filtered for power-line noise (60 Hz and its harmonics), and downsampled to 436 Hz.

### 2.5 Definition of events

Due to the self-paced nature of the experiment, and thus high variability in the reaction times (RT) across events and conditions, we decided to extract the signal as contiguous epochs of 1 s, considering each event onset as an integer number of seconds. As illustrated in Fig. 1B, each 1 s window was associated to a binary label (‘Math’ or ‘Non-math’) if a stimulus was the only stimulus present during the selected 1 s window. For this, we rounded each event onset to the closest integer (in s), and labeled the 1 s epochs corresponding to the duration of the event (as defined by the RT, rounded toward negative infinity) with the same category as the event. Each 1 s epoch was then considered as an individual trial. The four conditions of self-episodic, self-judgment, self-semantic and rest were pooled together as “non-math” (as in Dastjerdi et al., 2013) and equal number of “math” and “non-math” events was chosen in order to obtain balanced training sets.

### 2.6 Feature extraction

**Fig. 2:**
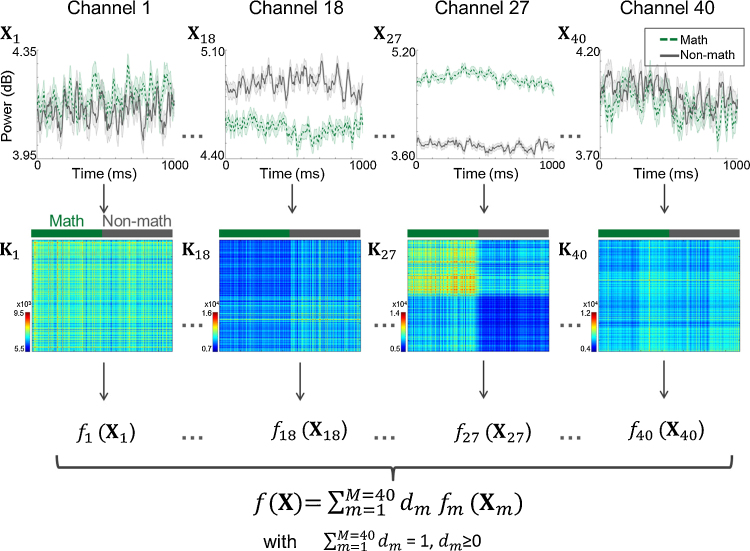
Illustration of the multiple kernel learning (MKL) approach considered. For each electrode, the time course of the power in a specific frequency band (here high-*γ*) is extracted for each trial in the [0 1 0 0 0] ms window around onset. The top row of the figure displays such features averaged across math (dashed green) and non-math (gray) trials (patient P1, session 1), with normalized standard error (shaded areas). From those features, a linear kernel is built for each electrode, as illustrated in the middle row of the figure (trials sorted by category, with Math trials in the top left corner). This matrix is symmetric and displays whether trials from one category are more similar to one another than to trials from another category (as is the case for kernels K_18_ and K_27_ but not for kernels K_1_ and K_40_). For each kernel, the model estimates a decision function *f_m_* (*m* = 1…*M*, *M* being the number of electrodes) which is then weighted according to a positive or null contribution *d*_m_. The final decision function is the linear combination of the different decision functions estimated on each electrode. The contributions of all electrodes must satisfy the constraints of summing to 1, and being positive or null, which leads to a sparse selection of electrodes contributing to the final model. (For interpretation of the references to color in this figure legend, the reader is referred to the web version of this article).

Each event was extracted (i.e., epoched) in the −200 ms – 1200 ms time window around its onset (in integer seconds, as defined above). No baseline correction or artifact rejection was performed such that the temporal correlation between epochs from the same event was maintained. A time-frequency decomposition was then computed (Morlet wavelets, 7 wavelets). The frequencies of interest were log-spaced between 1 and 110 Hz (29 values in total). The resulting decomposition was rescaled (point-wise) by the logarithm of its value. The instantaneous power of the signal in the 0 – 1000 ms time-window was then computed in each of the following frequency bands: *δ* (1–4 Hz), *θ* (4–8 Hz), *α* (8–12 Hz), *β* (15–25 Hz), low-*γ* (30–55 Hz) and a narrow band of HFB, high-*γ* (70–110 Hz, to avoid residual line noise), by averaging the frequency bins within those bands. Fig. 2 (top) displays such features averaged across categories (“math” in dashed green and “non-math” in grey) in the high-*γ* band for electrodes 1, 18, 27 and 40 in P1.

### 2.7 Kernels

The considered algorithm uses kernels as inputs. Kernels are matrices of size *n* × *n* (*n* being the number of samples/trials, here epochs), representing the pair-wise similarity between samples/trials. In the present work, linear kernels (i.e., dot product) were built for each electrode and each frequency band, as illustrated in Fig. 2. Thus *m* kernels were computed based on the averaged power of the signal within a specific frequency band on each channel, with *m* being the number of electrodes. The *m* × 6 kernels were then concatenated, to estimate what is further referred to as the ‘full’ model. To ensure that the scale of each kernel does not play a role in the modeling step, all kernels were mean-centered and normalized before entering the classification algorithm (by taking into account the train/test separation, see Section 2.9).

### 2.8 Machine learning modeling

All machine learning modeling steps were performed based on our software PRoNTo (Schrouff et al., 2013, www.mlnl.cs.ucl.ac.uk/pronto), which has been adapted to analyze electrophysiological data in the SPM MEEG format. The built kernels were considered for MKL modeling using the “simpleMKL” version of (Rakotomamonjy et al., 2008) (illustrated in Fig. 2). This algorithm uses a Support Vector Machine (SVM, (Cortes and Vapnik, 1995)) to define a decision boundary (*f*_m_), discriminating between “math” and “non-math” per kernel. To determine each decision boundary, model parameters (**w**_m_) are optimized. Those different decision boundaries (one per kernel) are then weighted (by a parameter *d*_m_) to define a global decision boundary *f*. These two steps are implemented into a recursive optimization procedure, and hence both the weights **w**_m_ and the kernel contributions *d*_m_ depend on all the features. The regularization constraints in the considered algorithm lead to a sparse selection of non-null contributions (*d*_m_) to the final decision function, i.e., only some kernels will have a non-null contribution to the decision function. The present model can be seen as being ‘hierarchical’, i.e., for each kernel it is possible to compute the weight, *w*_tm_, of each feature *t* in *f*_m_ (here 436 features, as for ‘classical’ machine learning techniques), and each kernel is weighted by a contribution *d*_m_. Information on the contribution of each feature is hence available. However, interpreting the contribution of the kernels might be easier as each kernel is built on specific dimensions, i.e., MKL ‘summarizes’ the contributions over the chosen dimension by automatically selecting all the features from this dimension with the same contribution *d*_m_ (but not with the same weight *w*_tm_). It is important to note that if those contributions *d*_m_ were equal for all kernels, the MKL technique would correspond to an SVM model (with model parameters **w**) considering all the features as concatenated.

In the present case, each kernel represents the power of the signal in one electrode, averaged within a frequency band (*δ*, *θ*, *α*, *β*, low-*γ* or high-*γ*). For each patient, the full model was estimated (*m* × 6 kernels). In addition, for comparisons with previous univariate results (Dastjerdi et al., 2013, Foster et al., 2012), an MKL model was estimated for each frequency band (i.e., with *m* kernels).

### 2.9 Cross-validation and accuracy

We assessed the performance of the model on the experimental condition by using a 10-fold cross-validation scheme: the model was trained using 90% of the events with their corresponding labels, and used to predict the labels of the 10% events left out. The predictions were then compared with the labels of those left out events to compute the accuracy for each category (“math” accuracy and “non-math” accuracy, a.k.a. the sensitivity), the balanced accuracy (average of the class accuracies) and the positive predictive values for each category (representing the specificity). This process was repeated 10 times, with a different split of 10% of the data left out each time. Each split of the data is further referred to as a ‘fold’. In this work, the accuracy presented was obtained by averaging the values over all folds.

The considered algorithm is based on SVM models, which includes a soft-margin parameter *C*. This hyperparameter penalizes more (large values of *C*) or less (small values of *C*) mis-classifications during training and affects the resulting decision boundary *f*. This is particularly the case in the present work since each kernel is based on 436 features (i.e., 1 electrode × 1 s time-window sampled at 436 Hz × averaged frequency band), and the number of events available for training is in the same order (368 for P1, 507 for P2 and 369 for P3). We therefore cannot expect the categories to be linearly separable as they would in a high-dimensional space. Values of *C* ranging from 0.01 to 1000 (*C* = 10^*i*^, *i* = −2, −1, …, 3) were hence considered. A nested cross-validation was performed when modeling the experimental conditions: the inner cross-validation selected the value of *C* leading to the highest model performance and the outer cross-validation estimated the performance on a test set using the selected value of *C*.

### 2.10 Statistical significance

The independence between the train and test sets is compromised due to temporal autocorrelation in the considered trials. In addition, different folds of the cross-validation are estimated based on overlapping training data (here as much as 80% of the data is shared between two folds). This hence precludes the use of any parametric tests such as binomial tests (Noirhomme et al., 2014, Pereira et al., 2009) to assess the statistical significance of our results. The significance of the performance of each model was assessed using 1000 permutations of the training labels. Results associated to a *p*-value smaller than 0.05 were reported as significant. Balanced accuracy values were considered as significant when both the classification accuracy for the “math” and “non-math” conditions were significant.

### 2.11 Localization of the kernel contributions

**Fig. 3:**
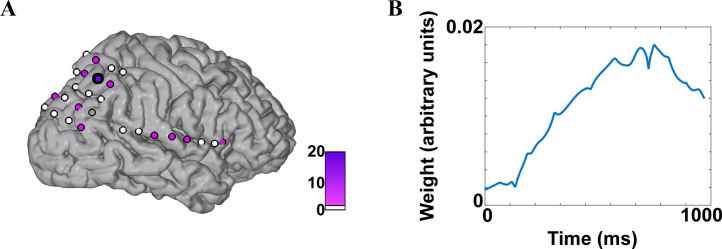
Illustration of the outputs of the MKL model. (A) Parietal view of the surface of the cortex of patient P1. Electrodes are displayed as circles on the cortex (not to scale) with a fill color corresponding to their contribution to the final model (*d*_m_, in %). (B) Weights **w**_m_ for the channel circled in black in panel A. These weights display the contribution of each time point from that channel to the MKL model. As the model is not sparse in terms of the **w**_m_, all the time points considered for modeling contribute to the final decision function.

Fig. 3 illustrates the outputs of the MKL model in the high- *γ* band for patient P1. Fig. 3A displays the kernel (here channel) contributions *d*_m_, while Fig. 3B displays the weights **w**_m_ for one channel in this frequency band.

#### 2.11.1 Sparseness of the results

As previously mentioned, the considered algorithm constrained the resulting kernel contribution (*d*_m_) to be sparse. Therefore, for each fold, a certain number of kernels – here corresponding to a combination of frequency and electrode (i.e., recording site) – had a non-null contribution to the final decision boundary. Because of the cross-validation, the contributions of each kernel were averaged across all folds (here 10). We hence investigated the stability of *d*_m_ across folds by counting how many kernels were consistently selected in all folds or in 80% of the folds (i.e., 8 folds out of 10 in the considered cross-validation scheme).

#### 2.11.2 Anatomical localization

In each frequency band, we looked at the contribution of each electrode (*d*_m_) to the model. This allowed localizing the information leading to the discrimination of “math” versus “non-math” in terms of recording site, i.e., anatomical position. For illustration, the contribution of electrodes (averaged across folds) was projected on the patient's 3D brain surface by color-coding the circle representing the anatomical position of each electrode. Fig. 3A displays the parietal view of the cortex of Patient P1, with channels with a white fill having a perfectly null contribution to the model and channels with a pink to purple fill contributing to the MKL model.

#### 2.11.3 Frequency localization

The full model allowed combining the information from different electrodes and different frequency bands, which helped us investigate whether the discriminating signal is localized in terms of frequency or, on the opposite, distributed across frequency bands. To this end, the *d*_m_ values corresponding to each frequency band were summed across electrodes, leading to a weight value per frequency band. These are represented on bar graphs for each patient.

#### 2.11.4 Temporal localization

As mentioned in the previous section, it would be possible to also investigate the weights *w*_tm_ for each time point of the time course of the power in each channel and frequency band pair. Those weights are displayed in Fig. 3B for one channel/frequency band combination (channel circled in black in Fig. 3A). As interpreting such weight values is complex and has recently raised various issues (see e.g., (Haynes, 2015) for a discussion on the topic), we have chosen to focus our interpretation of the results on the kernel contributions *d*_m_.

## 3 Results

### 3.1 Features and kernels

Each participating patient was implanted with different number of electrodes. After discarding electrodes containing noisy or pathological signals, different numbers of channels were selected for P1 (*n* = 40), P2 (*n* = 102) and P3 (*n* = 97). Hence, for each of the six frequency bands, 40, 102 and 97 kernels were computed for P1, P2 and P3, respectively. Therefore, the full model comprised 40 × 6 = 240 kernels for P1, 612 for P2 and 582 for P3.

### 3.2 Extraction of events

Regarding the two sessions of experimental condition, 184 math events were extracted for P1, 255 for P2 and 185 for P3. Numbers differ across participants because different numbers of events were rejected due to the presence of artifacts or epileptic activity in the signal. They were balanced with an equal number of epochs from each of the four other non-math conditions (self-episodic, self-judgment, self-semantic and rest conditions, random selection).

### 3.3 Model performance

Balanced and class accuracies estimated from the full model are displayed for each subject in Table 1. The model performance for each frequency band is also displayed for comparison with the univariate results (Dastjerdi et al., 2013).

For all subjects, the 6 frequency bands led to significant classification of math versus non-math. Classification accuracy was highest in the high-*γ* band for patients P1 (88.92%) and P2 (88.37%) while it was highest in the *α* band for patient P3 (75.03%). In addition, the full model showed similar accuracy to the accuracy obtained from the best band for each patient: P1 = 89.51%, P2 = 87.18% and P3 = 76.80%.

### 3.4 Localization of discriminating signal

#### 3.4.1 Sparseness of the results

**Fig. 4:**
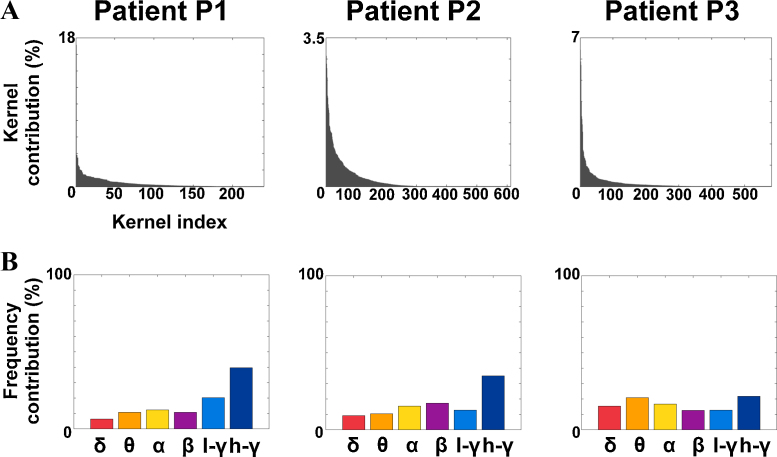
Sparseness and frequency localization of the kernel contributions, for each patient. (A) Sorted histogram of kernel contributions (averaged across folds). The displayed values sum to 100%, as constrained by the considered machine learning algorithm. (B) Summed contribution of each frequency band to the full model (averaged across folds), in percent.

The number of kernels with non-null contributions across all folds is presented in Table 2. The number of kernels consistently selected in 80 or in 100% of the folds are also displayed. Fig. 4A shows a histogram of the kernel contributions (averaged across folds), sorted in descending order, for each patient.

Table 2 shows that the results are sparse, but highly dependent on the training data, which is assumed to slightly vary from one fold of the cross-validation to another.

#### 3.4.2 Anatomical localization

**Fig. 5:**
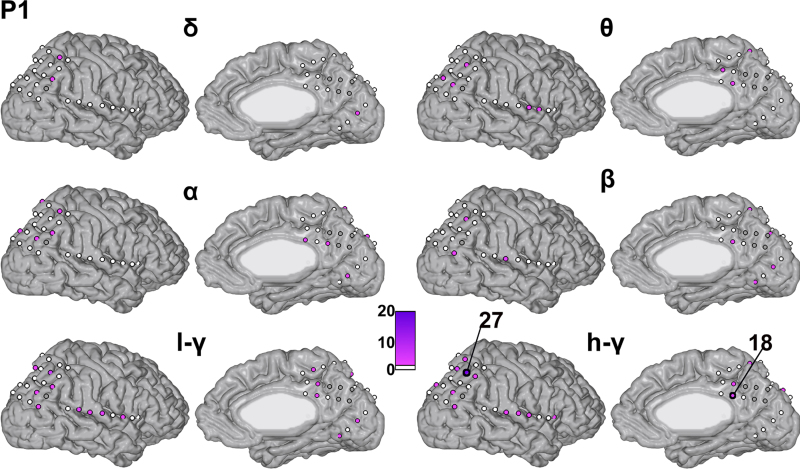
Channel contribution in each frequency band for patient P1. Projection of kernel contribution onto the electrode anatomical position on the patient's cortex (3D mesh), for each frequency band (l-*γ* stands for low-*γ* and h-*γ* for high-*γ*). Each circle on the cortex represents the position of an electrode (not to scale). The contribution of the corresponding kernel to the full model is then color-coded (see color bars): a white fill represents an electrode with a 0% contribution and the highest contributions are displayed in purple. Electrodes displayed with a grey fill were not considered for modeling (noisy/pathological electrodes). The electrode number of some electrodes – with a contribution ranked in the top 10 – is displayed for cross-reference with the text. (For interpretation of the references to color in this figure legend, the reader is referred to the web version of this article).

**Fig. 6:**
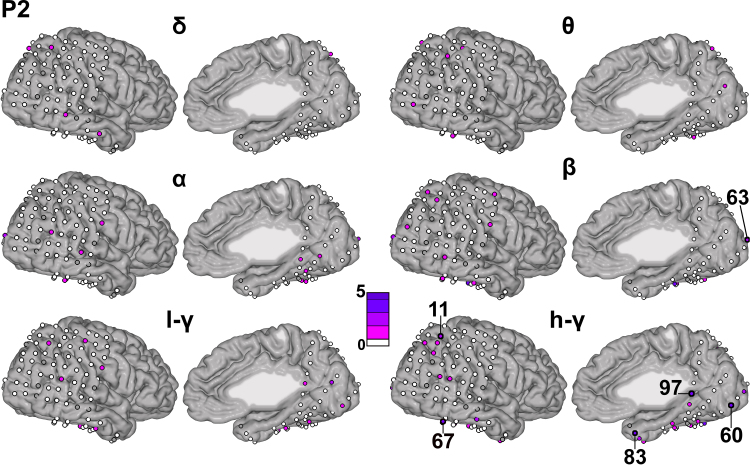
Channel contribution in each frequency band for patient P2.

**Fig. 7:**
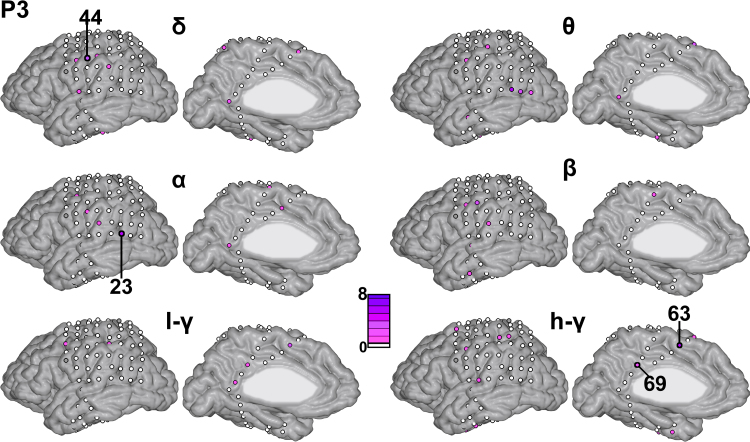
Channel contribution in each frequency band for patient P3.

Fig. 5, Fig. 6, Fig. 7 display the contribution of each kernel (i.e., each electrode in each of the considered frequency bands) for each patient, respectively. The electrodes are represented by circles (not to scale) on the patient's cortex, color-coded based on their contribution to the full model (average across folds). Table 3 displays the 10 electrode–frequency band combinations with the highest contribution to the full model for each patient.

For patient P1 (Fig. 5), electrode 27 had the largest contribution (*d*_m_ = 17.14% in the high-*γ* band and *d*_m_ = 3.81% in the low-*γ* band). This channel is located in the IPS. The electrode with the third highest contribution is channel 18 (3.47%), which is located in the RSC. The IPS electrode site is precisely the one reported in our previous publication to be highly activated during the math condition (Dastjerdi et al., 2013) and the RSC is the same site as the one reported in our previous work to be highly deactivated during the math condition (Foster et al., 2012). For patient P2 (Fig. 6), the distribution of weights is less sparse than for patient P1. The electrode with the largest contribution was electrode 97 in the high-*γ* band (3.40%), located in the RSC (i.e., the same site as the one reported in our previous work to be highly deactivated during the math condition, Foster et al., 2012). Among the other top 10 ranking sites (using averaged *d*_m_ for ranking) the temporal cortex (electrodes 83, 67, 84) as well as occipital cortex (electrodes 60, 62 and 63) and IPS (electrode 11) sites were marked as contributory. For P3 (Fig. 7), several electrodes (according to averaged *d*_m_) showed non-null contribution: 4 electrodes were in the lateral parietal cortex (highest: electrode 23, 5.88%, selected in the *θ* and *α* bands), as well as one electrode in the RSC (electrode 69, high-*γ* band). Non-null contributions were also found in the pre-supplementary motor region (electrode 63 in high-*γ*, 6.43%) and in the lateral parietal cortex (electrode 44 in *δ* and *β*).

#### 3.4.3 Frequency localization

When computing the total contribution of each frequency band to the full model, we observed that all frequency bands contributed to the model (Fig. 4C). For patients P1 and P2, the high-*γ* band had the largest contribution (39.7% for P1 and 35.0% for P2). For patient 3, the high-*γ* band accounted for 22% of the weights, the *θ* band for 21% and the *α* band for 17%.

## 4 Discussion

In this work, we present a multiple kernel learning machine based model to automatically select frequency and anatomical features discriminating between different experimental conditions. The proposed approach was data-driven and used the signal in three dimensions (i.e., evolution of the power in time and in each frequency band and in each electrode) to automatically determine which electrodes and/or frequency band contained discriminating information about numerical processing. The MKL technique can hence be seen as an exploratory technique to investigate electrophysiological signal on multiple dimensions simultaneously.

### 4.1 Comparison of univariate and multivariate results

The performed multivariate analyses led to significant classification of Math versus Non-math trials, using all electrodes and frequency bands as inputs. The highest accuracy values ranged between 77% and 89%. These values can hardly be directly compared to the sensitivity and specificity obtained using univariate techniques (Dastjerdi et al., 2013) since we obtained one value per subject and not one value per electrode. This can be seen as an advantage (i.e., ‘summarizing’ the results for each subject) or as a disadvantage (i.e., lack of localized information). More generally, univariate methods are thought to be more specific (i.e., localized detection of (de)activations) but less sensitive (more difficult to detect significant changes) than multivariate techniques. This last observation is supported in the present study as significant discrimination between Math and Non-math trials was observed in all frequency bands, which was not detected in the work of (Dastjerdi et al., 2013). We therefore consider that these two analyses investigate different aspects of the question of interest and should be used in conjunction rather than compared.

### 4.2 Information is distributed over frequency bands

When looking at the contribution of each frequency band to the full model, the results suggest that discriminating information is distributed across frequency bands. This is further supported by the fact that the full model may display even higher accuracy than a single frequency band even though the size of the input-space was multiplied by 6. In particular, the high-gamma, theta and alpha bands seemed to contain information about our variable of interest. This result is in line with previous modeling of ECoG data (van Gerven et al., 2013), which showed that ECoG signal had predictive power in a range of frequencies, especially in the 4–16 Hz and 65–128 Hz frequency bands.

In most of today's ECoG literature, there is a particular focus on the HFB. The present work shows that other frequency bands could also contain information about the variable of interest, either on their own or combined with other frequency bands (i.e., full model). This might bring further insights on how the information is coded in the human brain and might have important implications on upcoming studies based on ECoG recordings.

### 4.3 Automatic selection of features

During its estimation, the model automatically selected which electrodes and/or frequency bands should have a non-null contribution toward better discrimination between conditions. This data-driven analysis allowed us to identify the sites within the covered cortical regions that had been identified previously with univariate results. For instance, our sites with the largest contributions had been shown previously as significantly deactivated or activated during the math or non-math conditions (i.e., the IPS activation during math trials and RSC activations and deactivations during non-math and math trials, respectively (Dastjerdi et al., 2013, Foster et al., 2012). In line with the current ECoG literature, the high-*γ* band led to the highest modeling accuracy and had the largest weight in the full model for patients P1 and P2, which is also in agreement with our own previous univariate results (Dastjerdi et al., 2013). Univariate analyses however neglected to identify the significant contribution of *α* and *θ* bands as containing relevant information for patient P3, once more confirming the superior sensitivity of MKL machine learning model to identify distributed brain activity during a given cognitive condition.

### 4.4 Prospective uses

In the present work, the kernels were computed from the temporal evolution of the averaged power in a specific frequency band in each electrode. However, any feature could be considered for modeling (e.g., the raw signal on each channel, coherence values or phase values), depending on the clinical or cognitive neuroscience question to investigate. Moreover, the MKL approach as defined in this work can be transposed and applied to any combination of dimension, which allows investigating multiple aspects of such a question. For example, the signal in an epoch could be divided into sub-time windows of 10 ms, for each channel. The MKL model would hence provide information on the timing of best discrimination between two classes for each channel. This would allow investigating the temporal propagation of the signal of interest over the channels. In the same way, we used pre-defined frequency bands to illustrate our MKL approach. However, it might be of interest to consider subject-specific frequency bands (e.g., for *θ* activity). To this end, a kernel could be built for each frequency bin in a frequency band of interest. The resulting MKL model would then automatically select which frequency bins contain information about the variable of interest for this specific data set/subject. Finally, different types of features could be combined (e.g., power, phase and raw signal). The resulting MKL model would provide the contributions of each feature type to the final model. This would allow investigating whether different features bring complementary information regarding the variable of interest or not.

Furthermore, we chose to illustrate our method with ECoG data since the interpretation of the selection of electrodes in terms of anatomical location is straightforward. Practically however, the same technique can be applied to scalp EEG or MEG data.

Another prospective application of the MKL approach is that it allows investigating potential linear combinations between the signals on different electrodes, in the same or different frequency bands. This field of study recently received significant attention, especially when studying resting-state networks and how different conditions can affect the relationship between different areas within or between networks. In particular, phase-amplitude couplings could easily be investigated by computing one kernel per electrode using the power in a specific frequency band (e.g., HFB) and one kernel per electrode using the phase in the same or another frequency band (e.g., *θ*).

### 4.5 Limitations

It should be noted that the algorithm used in our present work (Rakotomamonjy et al., 2008) is sparse in terms of the contributions of the kernels to the final decision boundary. It is possible that the information of interest is more widely distributed across electrodes/frequency bands, and the considered algorithm may not select kernels containing correlated signals. As a result, neighboring electrodes are not often selected simultaneously in the same model estimation (only a subset is selected). Unfortunately, the considered formulation does not allow to control for the level of sparsity. If a non-sparse analysis is desired, one can use another type of regularization, such as the elastic-net (combination of L-1 and L-2 regularization, (Zou and Hastie, 2005)), as discussed in (van Gerven et al., 2013). Investigating MKL approaches with other types of regularizations is a topic for future work.

### 4.6 Availability of the method

The PRoNTo toolbox (Schrouff et al., 2013) has been adapted to perform the machine learning modeling of electrophysiological data (in the SPM MEEG format). All the functionalities presented in this work, as well as in the prospective uses have been implemented in the version 3.0 of our open-source software. PRoNTo v3.0 will be released as soon as possible (www.mlnl.cs.ucl.ac.uk/pronto).

### 4.7 Conclusion

In this work, we propose a sparse MKL approach to automatically select relevant sites of recording and/or frequency bands during cognitive processing in the human brain. We show that the MKL method is more sensitive than univariate methods to decode a variable of interest based on electrophysiological recordings. We also demonstrate that the MKL method provides an easy way to locate the information of interest in terms of anatomy, time window or frequency domain.

## Conflict of interest

None.

## Figures and Tables

**Table 1:** Model performance for each patient and model, displayed via balanced accuracy values and the sensitivity for “Math” and for “Non-Math” (in brackets, respectively). Those values represent averages over the 10 folds.

MKL model	Patient P1	Patient P2	Patient P3
*Full*	89.51 (88.06–90.96)	87.18 (85.49–88.89)	76.80 (72.89–80.71)
*δ*	73.48 (69.25–77.71)	82.25 (81.18–83.33)	72.36 (69.01–75.71)
*θ*	70.92 (68.66–73.16)	79.89 (78.43–81.35)	73.08 (69.37–76.79)
*α*	75.28 (71.64–78.92)	75.57 (71.37–79.76)	75.03 (70.77–79.29)
*β*	66.88 (64.18–69.58)	78.50 (78.04–78.97)	69.18 (65.14–73.21)
*low-γ*	85.61 (84.18–87.05)	81.07 (79.61–82.54)	68.81 (66.20–67.57)
*high-γ*	88.92 (86.27–86.86)	88.37 (87.45–89.29)	69.50 (69.37–69.64)

**Table 2:** Number of kernels with a non-null contribution to the full model, for each patient. The percentage of channels selected in the model compared to the total number of channels for each patient is displayed in brackets.

Number of kernels selected in	Patient P1	Patient P2	Patient P3
*At least one fold (average across folds)*	180 (75%)	309 (50%)	344 (59%)
*At least 80% of the folds*	59 (25%)	69 (11%)	25 (04%)
*All folds*	11 (05%)	36 (06%)	12 (02%)

**Table 3:** Top 10 ranking electrode-frequency band combinations for each patient, according to *d*_m_ averaged across folds.

Patient P1			Patient P2			Patient P3		
Band	Elec	*d*_m_ (%)	Band	Elec	*d*_m_ (%)	Band	Elec	*d*_m_ (%)
high-*γ*	27	17.14	high-*γ*	97	03.40	high-*γ*	63	06.43
low-*γ*	27	03.81	*β*	74	03.07	*α*	23	05.88
high-*γ*	18	03.47	high-*γ*	83	02.89	*θ*	23	05.71
high-*γ*	32	02.54	high-*γ*	67	02.78	*δ*	44	04.00
*β*	14	02.38	high-*γ*	60	02.56	*θ*	65	03.29
*α*	17	02.04	high-*γ*	62	02.13	*β*	44	03.17
low-*γ*	13	01.98	*α*	63	02.09	*θ*	16	02.91
high-*γ*	04	01.92	high-*γ*	84	01.93	low-*γ*	63	02.27
low-*γ*	15	01.68	*β*	63	01.85	high-*γ*	69	01.71
*θ*	20	01.47	high-*γ*	11	01.80	*δ*	66	01.67
